# Chemical Constituents of *Murraya tetramera* Huang and Their Repellent Activity against *Tribolium castaneum*

**DOI:** 10.3390/molecules22081379

**Published:** 2017-08-20

**Authors:** Chun-Xue You, Shan-Shan Guo, Wen-Juan Zhang, Zhu-Feng Geng, Jun-Yu Liang, Ning Lei, Shu-Shan Du, Zhi-Wei Deng

**Affiliations:** 1 Beijing Key Laboratory of Traditional Chinese Medicine Protection and Utilization, Faculty of Geographical Science, Beijing Normal University, No.19, Xinjiekouwai Street, Beijing 100875, China; youchunxue@mail.bnu.edu.cn (C.-X.Y.); ssdyx1990@163.com (S.-S.G.); zwj0729@mail.bnu.edu.cn (W.-J.Z.); liangjunyu@nwnu.edu.cn (J.-Y.L.); 2 College of Animal Science and Veterinary Medicine, Tianjin Agricultural University, Tianjin 300384, China; 3 Analytical and Testing Center, Beijing Normal University, Beijing 100875, China; gengzhufeng@bnu.edu.cn (Z.-F.G.); dengzw@bnu.edu.cn (Z.-W.D.); 4 Department of Pharmacy, The General Hospital of the PLA Rocket Force, Xicheng District, Beijing 100088, China

**Keywords:** *T. castaneum*, *M. tetramera*, repellent activity, grey relational analysis, chemical constituents

## Abstract

Sixteen compounds were isolated from the leaves and stems of *Murraya* *tetramera* Huang. Based on the NMR and MS spectral results, the structures were determined. It was confirmed that the isolated compounds included three new compounds (**9**, **10** and **13**) and one new natural product (**8**), which were identified asmurratetra A (**9**), murratetra B (**10**), murratetra C (**13**) and [2-(7-methoxy-2-oxochromen-8-yl)-3-methylbut-2-enyl]3-methylbut-2-enoate (**8**), respectively. Meanwhile, the repellent activity against *Tribolium castaneum* was investigated for 13 of these isolated compounds. The results showed that the tested compounds had various levels of repellent activity against *T. castaneum*. Among them, compounds **1** (4(15)-eudesmene-1β,6α-diol), **11** (isoferulic acid) and **16** (2,3-dihydroxypropyl hexadecanoate) showed fair repellent activity against *T. castaneum*. They might be considered as potential leading compounds for the development of natural repellents.

## 1. Introduction

The red flour beetle (*Tribolium castaneum*) is one of the most destructive pests in stored products and often causes serious losses of stored goods in warehouses of grains, foods or traditional Chinese medicinal materials [1,2]. In an infestation, *T. castaneum* not only consumes stored materials, but also leads to an accelerated growth of molds in elevated temperatures and humid environments [3]. Nowadays, synthetic insecticides are mainly used to control the insects in a warehousing system. However, widespread use of these chemicals has resulted in a series of problems such as pesticide residue, health hazards to humans, environmental pollution and insect resistance [4]. Today, the attention of more and more researchers has been focused on seeking plant-derived materials which could be used as effective natural anti-insect agents or leading compounds for the control of insects. Some successful substances have been found. For example, the commercial botanical insecticide pyrethrum is a natural mixture product extracted from *Chrysanthemum cinerariifolium*, and it has been widely used in the world [5,6].

The genus *Murraya* comprises about 12 species all over the world, and nine of them are widely distributed in the south China [7]. The plants possess a special aroma, and insect pests rarely appear on *Murraya* species. In practice, it has been reported that some plants of the genus possess anti-insect properties [8,9,10]. The resources of genus *Murraya* are abundant in our country; however, the utilization of genus *Murraya* resources is still inadequate. Except a few parts of plants such as *Murraya exotica*, *Murraya paniculata* being used as medicine, most of the plants still have not been used properly. If the active constituents for control of the stored product insects are found from the waste plant resources, as it was expected, it would provide a new pesticide and the resources of genus *Murraya* would be further exploited and used as well.

In our previous works, it was found that the methanol extract of *Murrayate tramera* Huang showed significant repellent activity. In this work, compounds with significant repellent activity were expected to be obtained from the methanol extract of *M. tetramera*. Here, three new compounds, one new natural product and 12 known compounds were isolated from the methanol extract of *M. tetramera* and their repellent activity was evaluated.

## 2. Results

### 2.1. Compounds Isolated from M. tetramera

**Figure 1:**
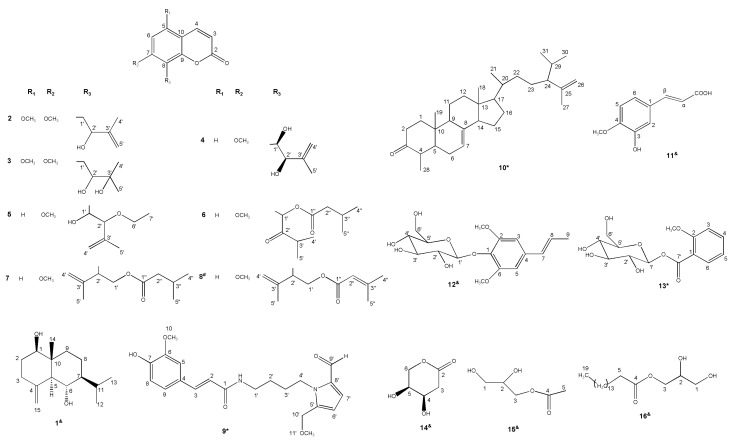
Chemical structures of compounds **1**–**16**; ^*^ represents new compound, ^#^ represents new natural product, ^&^ represents first isolated compound from *Murraya* genus.

Sixteen compounds were isolated from the leaves and stems of *M. tetramera* and their molecular structures were determined based on the MS (Mass Spectrometry) and NMR (Nuclear Magnetic Resonance) spectra. They included several kinds of compounds such as a sesquiterpenoid (**1**), coumarins (**2**–**8**), an amide (**9**), a triterpenoid (**10**), etc. Among them, there were three new compounds which were named murratetra A (**9**), murratetra B (**10**), murratetra C (**13**), respectively, and a new natural product which was confirmed as [2-(7-methoxy-2-oxochromen-8-yl)-3-methylbut-2-enyl]3-methylbut-2-enoate (**8**). The other compounds were identified as 4(15)-eudesmene-1β,6α-diol (**1**) [11], sibirinol (**2**) [12], mexoticin (**3**) [13,14], murrangatin (**4**) [15,16], 2’-*O*-ethylmurrangatin (**5**) [17], paniculatin (**6**) [18], isomurralonginol isovalerate (**7**) [19], isoferulic acid (**11**) [20], acantrifoside E (**12**) [21], 4(*R*), 5(*S*)-dihydroxy-tetrahydro-pyran-2-one (**14**) [22], 2,3-dihydroxypropyl acetate (**15**) [23], 2,3-dihydroxypropyl hexadecanoate (**16**) [24]. All their molecular structures are displayed in Figure 1.

### 2.2. Molecular Structural Elucidation of the New Compounds

**Figure 2:**
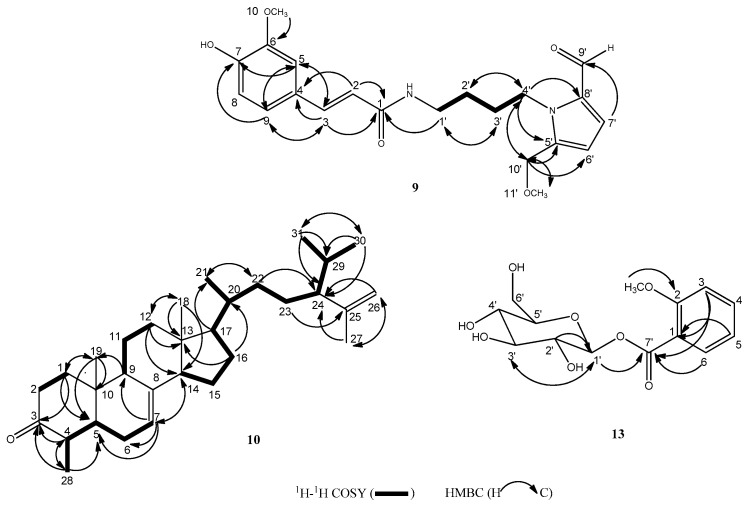
The key COSY and HMBC correlation signals of the new compounds.

Compound **9** was obtained as a pale yellow oil. The molecular formula was assigned as C_21_H_26_N_2_O_5_ by HR-ESI-MS (high resolution electrospray ionization mass spectrometry), which indicted an [M + H]^+^ peak at *m*/*z* 387.1912 (calculated for C_21_H_27_N_2_O_5_, 387.1914). In the ^13^C-NMR spectrum, 21 carbon signals were observed. The ^13^C-NMR data revealed that the tested compound had two carbonyl carbons at *δ*_C_ 179.7, *δ*_C_ 167.8, 12 olefinic carbons, five alkyl carbons and two methoxy groups. From the ^1^H-NMR spectrum, the presence of one aldehyde group at *δ*_H_ 9.47 (1H, s) was confirmed, as well as resonances for the protons of a typical ABX system at *δ*_H_ 7.14 (1H, d, *J* = 1.5 Hz), *δ*_H_ 7.05 (1H, dd, *J* = 1.5 Hz, 8.0 Hz), *δ*_H_ 6.82 (1H, d, *J* = 8.0 Hz), a pair of trans doublets at *δ*_H_ 7.45 (1H, d, *J* = 15.5 Hz), *δ*_H_ 6.45 (1H, d, *J* = 15.5 Hz) and two methoxy groups at *δ*_H_ 3.91 (3H, s), *δ*_H_ 3.36 (3H, s). A butyl group was deduced from the H-H COSY correlations between H-4’ (*δ*_H_ 4.38) and H-3’ (*δ*_H_ 1.80), and between H-2’ (*δ*_H_ 1.62) and H-1’ (*δ*_H_ 3.35), H-3’ (*δ*_H_ 1.80). The HMBC spectrum showed correlations arising from H-3 (*δ*_H_ 7.45) to C-5 (*δ*_C_ 110.0), C-9 (*δ*_C_ 121.8), C-4 (*δ*_C_ 126.8), C-1 (*δ*_C_ 167.8), H-5 (*δ*_H_ 7.14) to C-9 (*δ*_C_ 121.8), C-3 (*δ*_C_ 140.7), C-7 (*δ*_C_ 148.4), H-9 (*δ*_H_ 7.05) to C-5 (*δ*_C_ 110.0), C-3 (*δ*_C_ 140.7), C-7 (*δ*_C_ 148.4), H-7’ (*δ*_H_ 7.01) to C-9’ (*δ*_C_ 179.7), H-2 (*δ*_H_ 6.45) to C-4 (*δ*_C_ 126.8), C-1 (*δ*_C_ 167.8), H-10’ (*δ*_H_ 4.52) to C-4’ (*δ*_C_ 45.0), C-11’ (*δ*_C_ 56.8), C-6’ (*δ*_C_ 111.5), C-5’ (*δ*_C_ 139.6), H-4’ (*δ*_H_ 4.38) to C-2’ (*δ*_C_ 26.3), C-10’ (*δ*_C_ 65.0), C-8’ (*δ*_C_ 132.4), C-5’ (*δ*_C_ 139.6), H-10 (*δ*_H_ 3.91) to C-6 (*δ*_C_ 147.9), H-11’ (*δ*_H_ 3.36) to C-10’ (*δ*_C_ 65.0), H-1’ (*δ*_H_ 3.35) to C-3’ (*δ*_C_ 28.4), C-1 (*δ*_C_ 167.8), H-3’ (*δ*_H_ 1.80) to C-1’ (*δ*_C_ 38.6), H-2’ (*δ*_H_ 1.62) to C-4’ (*δ*_C_ 45.0). The H–H COSY and HMBC correlations are presented in Figure 2. On the basis of the results and literature [25], the structure of compound **9** was identified as (*E*)-*N*-(4-(2-formyl-5-(methoxymethyl)-1*H*-pyrrol-1-yl)butyl)-3-(4-hydroxy-3-methoxyphenyl)acrylamide.

Compound **10** was obtained as colorless needles. The molecular formula was assigned as C_31_H_50_O by HR-ESI-MS, which indicted an [M + H]^+^ peak at *m*/*z* 439.3932 (calculated for C_31_H_51_O, 439.3934). This was in agreement with the 31 carbon signals in the ^13^C-NMR. The ^13^C-NMR data revealed one carbonyl carbon at *δ*_C_ 213.5, four olefinic carbons at *δ*_C_ 147.4, *δ*_C_ 139.2, *δ*_C_ 117.1, *δ*_C_ 111.9, seven methyl carbons at *δ*_C_ 21.5, *δ*_C_ 20.9, *δ*_C_ 19.2, *δ*_C_ 18.9, *δ*_C_ 13.7, *δ*_C_ 11.9, *δ*_C_ 11.4, nine methylene carbons at *δ*_C_ 39.6, *δ*_C_ 39.4, *δ*_C_ 38.0, *δ*_C_ 34.1, *δ*_C_ 28.0, *δ*_C_ 27.9, *δ*_C_ 26.5, *δ*_C_ 22.9, *δ*_C_ 21.5, eight methine carbons at *δ*_C_ 55.9, *δ*_C_ 55.5, *δ*_C_ 54.8, *δ*_C_ 50.3, *δ*_C_ 49.2, *δ*_C_ 45.6, *δ*_C_ 36.7, *δ*_C_ 30.3. The ^1^H-NMR spectrum displayed three olefinic protons at *δ*_H_ 5.22 (1H, d, *J* = 4.0 Hz) due to a trisubstituted double bond and *δ*_H_ 4.76 (1H, s), *δ*_H_ 4.63 (1H, s) due to a terminal methylene. The ^1^H-NMR spectrum also showed three angular methyl groups at *δ*_H_ 1.59 (3H, s), *δ*_H_ 1.10 (3H, s), *δ*_H_ 0.58 (3H, s), and four secondary methyl group doublets at *δ*_H_ 1.03 (3H, d, *J* = 6.5 Hz), *δ*_H_ 0.96 (3H, d, *J* = 6.5 Hz), *δ*_H_ 0.93 (3H, d, *J* = 6.0 Hz), *δ*_H_ 0.83 (3H, d, *J* = 6.0 Hz). The H-H COSY spectrum exhibited the connections of H-1/H-2, H-28/H-4/H-5/H-6/H-7, H-9/H-11/H-12, and H-14/H-15/H-16/H-16/H-20/H-22 (H-21)/H-23/H-24/H-29/H-30 (H-31). The HMBC spectrum showed correlations arising from H-1 (*δ*_H_ 2.18, 1.49) to C-5 (*δ*_C_ 50.3), C-3 (*δ*_C_ 213.5), C-19 (*δ*_C_ 13.7), H-4 (*δ*_H_ 2.33) to C-3 (*δ*_C_ 213.5), C-28 (*δ*_C_ 11.4), H-7 (*δ*_H_ 5.22) to C-5 (*δ*_C_ 50.3), C-6 (*δ*_C_ 28.0), C-9 (*δ*_C_ 55.9), C-14 (*δ*_C_ 54.8), H-9 (*δ*_H_ 1.22) to C-19 (*δ*_C_ 13.7), H-12 (*δ*_H_ 2.06, 1.26) to C-13 (*δ*_C_ 43.3), C-14 (*δ*_C_ 54.8), C-18 (*δ*_C_ 11.9), H-14 (*δ*_H_ 1.83) to C-7 (*δ*_C_ 117.1), H-16 (*δ*_H_ 1.91, 1.27) to C-13 (*δ*_C_ 43.3), C-20 (*δ*_C_ 36.7), H-17 (*δ*_H_ 1.75) to C-21 (*δ*_C_ 18.9), H-18 (*δ*_H_ 0.58) to C-12 (*δ*_C_ 39.4), C-13 (*δ*_C_ 43.3), C-14 (*δ*_C_ 54.8), H-19 (*δ*_H_ 1.10) to C-1 (*δ*_C_ 39.6), C-5 (*δ*_C_ 50.3), C-10 (*δ*_C_ 35.1), H-21 (*δ*_H_ 0.83) to C-22 (*δ*_C_ 34.1), H-22 (*δ*_H_ 1.35, 0.86) to C-21 (*δ*_C_ 18.9), C-24 (*δ*_C_ 55.5), H-23 (*δ*_H_ 1.61, 1.26) to C-25 (*δ*_C_ 147.4), H-26 (*δ*_H_ 4.76, 4.63) to C-24 (*δ*_C_ 55.5), C-27 (*δ*_C_ 19.2), H-27 (*δ*_H_ 1.59) to C-24 (*δ*_C_ 55.5), C-26 (*δ*_C_ 111.9), H-28 (*δ*_H_ 1.03) to C-3 (*δ*_C_ 213.5), C-4 (*δ*_C_ 45.6), C-5 (*δ*_C_ 50.3), H-30 (*δ*_H_ 0.96) to C-24 (*δ*_C_ 55.5), C-29 (*δ*_C_ 30.3), C-31 (*δ*_C_ 20.9), H-31 (*δ*_H_ 0.93) to C-24 (*δ*_C_ 55.5), C-29 (*δ*_C_ 30.3), C-30 (*δ*_C_ 21.5). The H-H COSY and HMBC correlations are presented in Figure 2. On the basis of the results and literature [26], the structure of compound **10** was identified as 17-(5-isopropyl-6-methylhept-6-en-2-yl)-4,10,13-trimethyl-4,5,6,9,10,11,12,13,14,15,16,17-dodecahydro-1*H*-cyclopenta[*a*]phenanthren-3(2*H*)-one.

Compound **13** was obtained as a yellow oil. The molecular formula was assigned as C_14_H_18_O_8_ by HR-ESI-MS, which indicted an [M + Na]^+^ peak at *m*/*z* 337.0916 (calculated for C_14_H_18_NaO_8_, 337.0894). This was verified by 14 carbon signals in the ^13^C NMR. The ^13^C-NMR data revealed one carbonyl carbon at *δ*_C_ 157.3, six olefinic carbons, one of them bearing a methoxy group at *δ*_C_ 167.2, six characteristic peaks of a glucose, and one methoxy group. The ^1^H-NMR spectrum displayed one *ortho*-disubstituted phenyl moieties at *δ*_H_ 7.78 (1H, d, *J* = 8.0 Hz), *δ*_H_ 7.56 (1H, t, *J* = 8.0 Hz), *δ*_H_ 7.42 (1H, d, *J* = 8.0 Hz), *δ*_H_ 7.15 (1H, t, *J* = 8.0 Hz), as well as the seven characteristic peaks of a glucose, including the terminal proton at *δ*_H_ 4.91 (1H, d, *J* = 7.5 Hz), and one methoxy group. The HMBC spectrum showed correlations arising from H-6 (*δ*_H_ 7.78) to C-7’ (*δ*_C_ 157.3), H-3 (*δ*_H_ 7.42) to C-1 (*δ*_C_ 121.0), C-7’ (*δ*_C_ 157.3), H-5 (*δ*_H_ 7.15) to C-1 (*δ*_C_ 121.0), H-1’ (*δ*_H_ 4.91) to C-3’ (*δ*_C_ 77.1), C-7’ (*δ*_C_ 157.3), 2-OCH_3_ (*δ*_H_ 3.91) to C-2 (*δ*_C_ 167.2), H-2’ (*δ*_H_ 3.53) to C-1’ (*δ*_C_ 102.7), H-3’ (*δ*_H_ 3.47) to C-1’ (*δ*_C_ 102.7). The HMBC correlations are presented in Figure 2. On the basis of the results and literature [27], the structure of compound **13** was identified as 2-methoxy-benzoyl-*β*-d-glucopyranoside.

### 2.3. Characterization of Isolated Compounds

*2-(7-Methoxy-2-oxochromen-8-yl)-3-methylbut-2-enyl] 3-methylbut-2-enoate* (**8**). Yellow oil. ^1^H-NMR (500 MHz, CDCl_3_) *δ* ppm: 7.64 (1H, d, *J* = 9.5 Hz, H-4), 7.37 (1H, d, *J* = 8.5Hz, H-5), 6.87 (1H, d, *J* = 8.5Hz, H-6), 6.27 (1H, d, *J* = 9.5Hz, H-3), 5.58 (1H, s, H-2”), 4.94 (1H, s, H-4’a), 4.93 (1H, s, H-4’b), 4.87 (1H, dd, *J* = 7.0Hz, 10.5Hz, H-1’a), 4.64 (1H, dd, *J* = 7.0 Hz, 10.5 Hz, H-1’b), 4.53 (1H, t, *J* = 7.0 Hz, H-2’), 3.89 (3H, s, 7-OCH_3_), 2.07 (3H, s, H-5”), 1.85 (3H, s, H-4”); 1.73 (3H, s, H-5’). ^13^C-NMR (125 MHz, CDCl_3_) *δ* ppm: 166.7 (C-1”), 161.2 (C-7), 160.8 (C-2), 156.2 (C-3”), 153.6 (C-9), 143.8 (C-4), 142.7 (C-3’), 127.5 (C-5), 116.6 (C-8), 116.2 (C-2”), 113.2 (C-3), 113.1 (C-10), 111.7 (C-4’), 107.9 (C-6), 63.8 (C-1’), 56.1 (7-OCH_3_), 40.7 (C-2’), 27.4 (C-4”), 22.3 (C-5’), 20.1 (C-5”).

*(E)-N-(4-(2-Formyl-5-(methoxymethyl)-1H-pyrrol-1-yl) butyl)-3-(4-hydroxy-3-methoxyphenyl) acrylamide* (**9**). Pale yellow oil. ^1^H-NMR (500 MHz, CD_3_OD) *δ* ppm: 9.47 (1H, s, H-9’), 7.45 (1H, d, *J* = 15.5Hz, H-3), 7.14 (1H, d, *J* = 1.5Hz, H-5), 7.05 (1H, dd, *J* = 1.5Hz, 8.0 Hz, H-9), 7.01 (1H, d, *J* = 4.0 Hz, H-7’), 6.82 (1H, d, *J* = 8.0 Hz, H-8),6.45 (1H, d, *J* = 15.5Hz, H-2), 6.31 (1H, d, *J* = 4.0 Hz, H-6’), 4.52 (2H, s, H-10’), 4.38 (2H, t, *J* = 8.0 Hz, H-4’), 3.91 (3H, s, 10-OCH_3_), 3.36 (3H, s, 11’-OCH_3_),3.35 (2H, t, *J* = 8.0 Hz, H-1’), 1.80 (2H, m, H-3’), 1.62 (2H, m, H-2’). ^13^C-NMR (125 MHz, CD_3_OD) *δ* ppm: 179.7 (C-9’), 167.8 (C-1), 148.4 (C-7), 147.9 (C-6), 140.7 (C-3), 139.6 (C-5’), 132.4 (C-8’), 126.8 (C-4), 124.5 (C-7’), 121.8 (C-9), 117.2 (C-2), 115.0 (C-8), 111.5 (C-6’), 110.0 (C-5), 65.0 (C-10’), 56.8 (C-11’), 54.9 (C-10), 45.0 (C-4’), 38.6 (C-1’), 28.4 (C-3’), 26.3 (C-2’).

*17-(5-Isopropyl-6-methylhept-6-en-2-yl)-4,10,13-trimethyl-4,5,6,9,10,11,12,13,14,15,16,17-dodeca hydro-1H-cyclopenta[a]phenanthren-3(2H)-one* (**10**). Colorless needles. ^1^H-NMR (500 MHz, CDCl_3_) *δ* ppm: 5.22 (1H, d, *J* = 4.0 Hz, H-7), 4.76 (1H, s, H-26a), 4.63 (1H, s, H-26b), 2.50 (1H, m, H-2a), 2.33 (1H, m, H-4), 2.30 (1H, m, H-2b), 2.18 (1H, m, H-1a), 2.15 (1H, m, H-6a), 2.06 (1H, m, H-12a), 1.91 (1H, m, H-16a), 1.83 (1H, m, H-14), 1.81 (1H, m, H-6b), 1.75 (1H, m, H-17), 1.62 (2H, m, H-11), 1.61 (1H, m, H-23a), 1.59 (3H, s, H-27), 1.55 (1H, m, H-24), 1.53 (1H, m, H-29), 1.49 (1H, m, H-1b), 1.45 (1H, m, H-5), 1.42 (2H, m, H-15), 1.35 (1H, m, H-22a), 1.34 (1H, m, H-20), 1.27 (1H, m, H-16b), 1.26 (1H, m, H-23b), 1.26 (1H, m, H-12b), 1.22 (1H, m, H-9), 1.10 (3H, s, H-19), 1.03 (3H, d, *J* = 6.5 Hz, H-28), 0.96 (3H, d, *J* = 6.5 Hz, H-30), 0.93 (3H, d, *J* = 6.0 Hz, H-31), 0.86 (1H, m, H-22b), 0.83 (3H, d, *J* = 6.0z, H-21), 0.58 (3H, s, H-18). ^13^C-NMR (125 MHz, CDCl_3_) *δ* ppm: 213.5 (C-3), 147.4 (C-25), 139.2 (C-8), 117.1 (C-7), 111.9 (C-26), 55.9 (C-9), 55.5 (C-24), 54.8 (C-14), 50.3 (C-5), 49.2 (C-17), 45.6 (C-4), 43.3 (C-13), 39.6 (C-1), 39.4 (C-12), 38.0 (C-2), 36.7 (C-20), 35.1 (C-10), 34.1 (C-22), 30.3 (C-29), 28.0 (C-6), 27.9 (C-16), 26.5 (C-23), 22.9 (C-15), 21.5 (C-11), 21.5 (C-30), 20.9 (C-31), 19.2 (C-27), 18.9 (C-21), 13.7 (C-19), 11.9 (C-18), 11.4 (C-28).

*2-Methoxy-benzoyl-β-d-glucopyranoside* (**13**). Yellow oil. ^1^H-NMR (500 MHz, CD_3_OD) *δ* ppm: 7.78 (1H, d, *J* = 8.0 Hz, H-6), 7.56 (1H, t, *J* = 8.0 Hz, H-4), 7.42 (1H, d, *J*= 8.0 Hz, H-3), 7.15 (1H, t, *J* = 8.0 Hz, H-5), 4.91 (1H, d, *J* = 7.5 Hz, H-1’), 3.93 (1H, m, H-6’a), 3.91 (3H, s, -OCH_3_), 3.74 (1H, m, H-6’b), 3.53 (1H, m, H-2’), 3.50 (1H, m, H-5’), 3.47 (1H, m, H-3’), 3.43 (1H, m, H-4’). ^13^C-NMR(125MHz, CD_3_OD) *δ* ppm: 167.2 (C-2), 157.3 (C-7’), 133.7 (C-4), 130.7 (C-6), 122.3 (C-5), 121.0 (C-1), 117.6 (C-3), 102.7 (C-1’), 77.1 (C-3’), 76.1 (C-5’), 73.6 (C-2’), 69.9 (C-4’), 61.2 (C-6’), 56.1 (2-OCH_3_).

### 2.4. Repellent Activity

The repellent activity against *T. castaneum* was investigated for 13 isolated compounds including the new compound (**10**), using DEET (*N*,*N*-diethyl-3-methylbenzamide) as a positive control sample. The results are presented in Table 1.

The results showed that the isolated compounds exhibited various levels of repellent activity against *T. castaneum.* Compared with the positive control, DEET, compounds **1**, **11**, **16** exhibited significant repellent activity against *T. castaneum.* At the concentrations of 78.63, 3.15, and 0.63 µg/cm^2^, compound **1** and DEET (*p* = 0.901, 0.990, 0.887) exhibited the same level of repellency against *T. castaneum* at 2 h after exposure. At the concentrations of 78.63, 15.73, 3.15, and 0.63 µg/cm^2^, compound **1** and DEET (*p* = 1.000, 0.914, 0.998, 0.289) exhibited the same level of repellency against *T. castaneum* at 4 h after exposure. Moreover, at the concentration of 0.13 µg/cm^2^, compound **1** exhibited stronger repellency than DEET (*p* = 0.000 and 0.000) against *T. castaneum* at 2 and 4 h after exposure. At the concentrations of 0.63 µg/cm^2^, compound **11** and DEET (*p* = 1.000) exhibited the same level of repellency against *T. castaneum* at 2 h after exposure. At the concentrations of 78.63, 15.73, 3.15, and 0.63 µg/cm^2^, compound **11** and DEET (*p* = 0.996, 0.775, 1.000, 1.000) exhibited the same level of repellency against *T. castaneum* at 4 h after exposure. Moreover, at the concentration of 0.13 µg/cm^2^, compound **11** exhibited stronger repellency than DEET (*p* = 0.000) against *T. castaneum* at 2 h after exposure. At the concentrations of 15.73, 3.15, and 0.63 µg/cm^2^, compound **16** and DEET (*p* = 0.227, 0.274, 0.996) exhibited the same level of repellency against *T. castaneum* at 2 h after exposure. At the concentrations of 78.63, 15.73, 3.15, and 0.63 µg/cm^2^, compound **16** and DEET (*p* = 0.542, 0.997, 0.971, 0.306) exhibited the same level of repellency against *T. castaneum* at 4 h after exposure.

## 3. Discussion

There were not many research works with the chemical components of *M. tetramera* and bioactivities reported. However, some papers have revealed some interesting chemical information about *M. tetramera*. For example, two new carbazole dimers, two novel heterodimers and three known analogues of carbazole were isolated from *M. tetramera* and some of them exhibited inhibition effects on NO production in BV-2 microglial cells [29,30], and You reported that two sesquiterpenes and three coumarins isolated from *M. tetramera* showed fair cytotoxic effects [31].

In this report, three new compounds and one new natural product were isolated from the methanol extract of *M. tetramera*, along with 12 other known compounds. The structures of three new compounds were determined with modern spectral techniques and they belonged to amide, triterpenoid and glucoside, respectively. The above work enriches the chemical information on *M. tetramera*. Although the plant of *M. tetramera* was historically used for insect prevention only few papers have been published about the chemical basis of insect prevention. Here the repellent activity against *T. castaneum* was primarily investigated for the chemical components isolated from *M. tetramera*. The results indicated that the variety of repellent activities between the compounds was obviously affected by the testing concentrations and the exposure duration. By comparing the repellent levels of five different concentrations, we found the recommended concentration of compounds **1** and **11** against *T. castaneum* was 78.63 µg/cm^2^, whereas 15.73 µg/cm^2^ is the recommended concentration of compound **16** against *T. castaneum*. The above findings indicated that the compounds **1**, **11** and **16** have the potential for further development as possible natural repellents for the control of insects in stored products. However, to develop a practical application for the pure compounds as novel repellents, further investigations that focus on the efficiency and safety of the compounds should be conducted. Moreover, additional studies on the development of formulations are also needed to improve the efficacy and stability.

The various repellent activities were related to the different chemical structures. Among the 13 compounds, compound **1** showed the most potent repellent activity against *T. castaneum*. The significant repellent activity of the sesquiterpenoid was related to its volatility. Some sesquiterpenoids in the literature also indicated fair repellent activity against *T. castaneum* [32,33,34]. Coumarins **3**–**6** shared the same basic skeleton with different substitution patterns, yet their repellent activities varied greatly. Interestingly, the results showed that the compounds possessing carbonyls on 8-substituents had weak repellent activity. However, compounds possessing double bonds on 8-substituents had better repellent activity. Thus, we conjectured the carbonyls and double bonds on 8-substituents of coumarins were related to the repellent activity. Compounds **3**–**6** shared a similar basic skeleton, yet their repellent activities varied greatly. The results indicated the repellent activity of ester compounds decreased after ring closing and a longer alkyl-substituent contributed to the repellent activity. Further study is needed to investigate the structure-active relationships. The yields of pure compounds were low, so the application was limited. The structure modification and synthesis of the compounds are considerable methods.

In addition, the plant species had already been tested with the essential oils [33]. Compared with the methanol extract, the essential oil exhibited stronger repellency against *T. castaneum*. The *PR* value averages were assigned to different classes (0 to V) in the literature using the following scale [35]. Class, *PR* value%: 0, 0.01–0.1; I, 0.1–20.0; II, 20.1–40.0; III, 40.1–60.0; IV,60.1–80.0; and V, 80.1–100. At the concentration of 78.63 nL/cm^2^, the *PR* value of the essential oil was 98% (class V) at 2 h after exposure and 100% (class V) at 4 h after exposure. At the concentration of 15.73 nL/cm^2^, the *PR* value of the essential oil was 94% class V) at 2 h after exposure and 100% (class V) at 4 h after exposure. At the concentration of 314.54 nL/cm^2^, the *PR* value of the methanol extract was 83% at 2 h after exposure and 93% (class V) at 4 h after exposure. At the concentration of 62.91 nL/cm^2^, the *PR* value of the methanol extract was 67% (class IV) at 2 h after exposure and 80% (class IV) at 4 h after exposure. The compounds isolated from the essential oil exhibited significant repellent activity against *T. castaneum*. The *PR* values of α-terpinene, β-caryophyllene, spathulenol, α-caryophyllene were greater than 80.1% (class V), and the *PR* values of alloaromadendrene, β-eudesmol were 80% (class IV) at the testing concentration of 78.63 nL/cm^2^ and 2 h after the insects were exposed. However, compounds **1**, **11**, **16** isolated from the methanol extract also exhibited strong repellent activity against *T. castaneum*. The *PR* values of the three compounds were greater than 80.1% (class V) at the testing concentration of 78.63 µg/cm^2^ and 2 h after exposure. The essential oil would be the better substance in terms of pest control. However, some compounds isolated from the methanol extract are also a choice for pest control.

## 4. Materials and Methods

### 4.1. General Information

NMR spectra were recorded on a Bruker Avance III NMR spectrometer with the magnetic field of 11.74 Tesla. HR-ESI-MS were acquired on a Bruker Q-TOF mass spectrometer. Preparative HPLC was performed on a Waters Delta Prep 4000 system equipped with a Waters 2487 dual λ absorbance detector. A Rainbow Kromasil-C_18_ (10 mm × 250 mm, 10 μm) column was selected for preparative HPLC. Column chromatography was performed on silica gel (160–200 mesh) and TLC analysis was carried on silica gel G plates (Qingdao Marine Chemical Plant, China). Sephadex LH-20 was purchased from Amersham Pharmacia Biotech (Beijing, China). MCI GELCHP20P (75–150 μm) was supplied by the Kaiteki Company (Tokyo, Japan). Analytical grade solvents were produced by Beijing Chemical Factory (Beijing, China).The deuterated solvents (CDCl_3_, CD_3_OD, DMSO-*d*_6_, deuterated ratio, 99.8%) with TMS as the internal referent were produced by Cambridge Isotope Labo-ratories, Inc. (Andover, MA, USA).

### 4.2. Plant Material

The leaves and stems of *M. tetramera* were collected in May 2014 from Xishuangbanna, Yunnan Province, China (21.13°–22.60°N latitude, 99.93°–101.83°E longitude). The plant was identified by Dr. Liu, Q.R. (College of Life Sciences, Beijing Normal University, Beijing, China) and the voucher specimen (BNU-CMH-Dushushan-2014-05-025-001) was deposited at the Herbarium (BNU) of College of Resources Sciences, Beijing Normal University (Beijing, China).

### 4.3. Insects

Laboratory cultures of the red flour beetles were maintained in the dark cabinet of an incubator at 28–30 °C and 70–80% relative humidity. The insects were reared in glass containers (0.5 L) containing wheat flour at 12–13% moisture content mixed with yeast (10:1, w/w). Insects used in the experiments were about seven-day-old adults.

### 4.4. Extraction and Isolation

**Figure 3:**
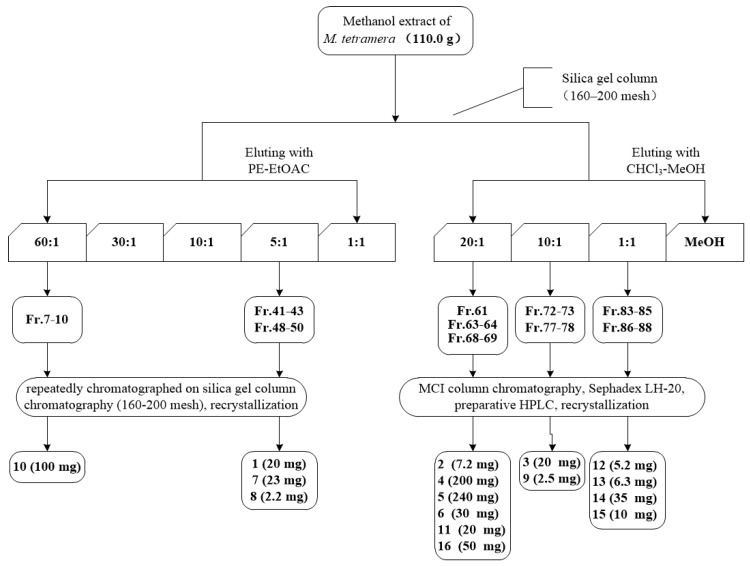
The tree-type figure for separation procedure.

Referring to the separation procedure of petroleum ether-ethyl acetate extract of *M. tetramera* [31], we isolated compounds from the methanol extract. The dried leaves and stems (2.5 kg) of *M. tetramera* were extracted three times with CH_3_OH (20 L) under ultrasound. The solvent was evaporated under vacuum to obtain the crude extract (110.0 g). The extract was fractionated by silica gel column chromatography (160–200 mesh, 10.0 × 33 cm, 1000 g), eluting witha stepwise gradient of PE/EtOAc (60:1, 30:1, 10:1, 5:1and 1:1), then CHCl_3_/CH_3_OH (20:1, 10:1, 1:1 and MeOH) to receive 90 fractions. Fractions with similar TLC spots were combined. Fr. 7–10 (1.63 g), Fr.41–43 (2.26 g), and Fr. 48–50 (3.17 g) were repeatedly chromatographed on silica gel column chromatography eluting with different polarity of PE/EtOAc to obtain compound **1** (20 mg), compound **7** (23 mg), compound **8** (2.2 mg) and compound **10** (100 mg), respectively. Fr. 61 (1.67 g), Fr. 63–64 (3.58 g), Fr. 68–69 (2.26 g), Fr. 72–73 (2.76 g), Fr. 77–78 (1.19 g), Fr. 83–85 (1.76 g) and Fr. 86–88 (1.29 g) were subjected to MCI column chromatography eluting with different polarity of EtOH, and then further isolated and purified by preparative HPLC using a mobile phase of MeOH-H_2_O to afford compound **2** (7.2 mg), compound **3** (20 mg), compound **4** (200 mg), compound **5** (240 mg), compound **6** (30 mg), compound **9** (2.5 mg), compound **11** (20 mg), compound **12** (5.2 mg), compound **13** (6.3 mg), compound **14** (35 mg), compound **15** (10 mg), and compound **16** (50 mg), respectively. The tree-type figure for separation procedure are presented in Figure 3.

### 4.5. Repellency Tests

The repellent activity of isolated compounds from *M. tetramera* against *T. castaneum* adults was tested using the area preference method [36]. The compounds were dissolved in ethanol to prepare testing solutions of five concentrations (78.63, 15.73, 3.15, 0.63, and 0.13 µg/cm^2^, respectively). The filter paper (9 cm in diameter) was cut into two equal pieces. One piece was uniformly treated with 500 μL of testing solution as a testing part, and the other piece was dealt with 500 μL of ethanol as a control part. The filter papers were air dried for about 8 min to evaporate the solvent completely, and then the two pieces of filter paper were tightly fixed on the bottom of a Petri dish side by side with solid glue. For each test, 20 insects were released at the center of the filter paper disc which was then covered with a lid. Five replicates were performed for each tested concentration and the experiment was repeated three times. The number of insects presented on the control (N_c_) and the testing (N_t_) parts of the filter paper wasrecorded after 2 and 4 h, respectively. A commercial repellent, *N,N*-diethyl-3-methylbenzamide (DEET, product of Dr. Ehrenstorfer, Germany), was used as a positive control. The value of percent repellency (*PR*) was calculated as follows:

*PR* (%) = [(N_c_ − N_t_) / (N_c_ + N_t_)] × 100

Analysis of variance (One-Way ANOVA) and Tukey’s test were conducted by using SPSS 20.0 (IBM Corp., Armonk, NY, USA) for Windows 2016 [37]. Percentage was subjected to an arcsine square-root transformation before variance and Tukey’s tests.

## 5. Conclusions

In this work, we investigated the chemical constituents of *Murraya tetramera* Huang. Three new compounds (**9**, **10**, **13**), one new natural product (**8**) and 12 known compounds (**1**–**7**, **11**, **12**, **14**–**16**) were isolated from the leaves and stems of *M. tetramera* Huang for the first time. The repellent activity of 13 compounds which were isolated from the *M. tetramera* was measured. Comparable to that of the positive control, DEET, compounds **1**, **11** and **16** exhibited significant repellent activity against *T. castaneum*. The results indicated that compounds **1**, **11** and **16** have potential for development into novel repellents to supply or substitute the heavy application of conventional repellents. They might be considered as potential leading compounds for the development of natural repellents. Further investigations that focus on the efficiency, safety and application of the compounds are also necessary.

## Figures and Tables

**Table 1:** Repellent activity of isolated compounds from *M. tetramera* against *T. castaneum*.

Treatment	PR% (Mean ± SE)									
	Concentration (µg/cm^2^)									
	2 h					4 h				
	78.63	15.73	3.15	0.63	0.13	78.63	15.73	3.15	0.63	0.13
Compound **1**	96 ± 4 ab ^**^	82 ± 7 bc	76 ± 9 ab	80 ± 6 a	70 ± 8 a	94 ± 7 a	76 ± 9 abc	74 ± 3 ab	70 ± 8 a	60 ± 8 a
Compound **3**	88 ± 7 b	64 ± 9 cd	−92 ± 9 f	−84 ± 12 e	−72 ± 12 f	80 ± 10 ab	56 ± 7 cd	−74 ± 9 e	−52 ± 4 d	−66 ± 7 f
Compound **4**	56 ± 7 c	50 ± 6 de	52 ± 7 bcd	46 ± 9 b	40 ± 8 bc	48 ± 9 c	58 ± 7 bcd	56 ± 9 ab	52 ± 7 ab	48 ± 9 ab
Compound **5**	54 ± 4 c	50 ± 6 de	64 ± 9 abc	58 ± 7 ab	30 ± 6 c	56 ± 9 bc	46 ± 4 d	62 ± 7 ab	64 ± 9 a	40 ± 6 abcd
Compound **6**	−30 ± 8 d	−34 ± 9 f	−20 ± 10 e	−26 ± 9 d	22 ± 7 cd	−42 ± 7 c	−58 ± 9 bcd	−16 ± 7 d	−20 ± 8 c	20 ± 6 d
Compound **10**	46 ± 9 c	40 ± 8 de	34 ± 7 cd	58 ± 7 ab	64 ± 7 ab	42 ± 7 c	50 ± 8 d	26 ± 4 c	32 ± 7 b	30 ± 8 bcd
Compound **11**	88 ± 7 b	82 ± 9 bc	66 ± 7 abc	62 ± 7 ab	38 ± 9 bc	92 ± 7 a	74 ± 9 abc	64 ± 9 ab	58 ± 7 a	46 ± 7 abc
Compound **14**	32 ± 7 c	30 ± 6 e	22 ± 4 d	16 ± 9 c	18 ± 7 cd	46 ± 9 c	16 ± 4 e	14 ± 7 c	−18 ± 7 c	−10 ± 8 e
Compound **15**	50 ± 6 c	46 ± 9 de	42 ± 9 cd	−26 ± 9 d	−16 ± 9 e	54 ± 7 bc	48 ± 7 d	52 ± 7 b	−30 ± 8 c	−10 ± 6 e
Compound **16**	86 ± 7 b	90 ± 6 ab	62 ± 7 abc	58 ± 7 ab	24 ± 7 cd	86 ± 9 a	80 ± 6 ab	76 ± 7 a	70 ± 6 a	22 ± 7 cd
DEET *	100 ± 0 a	98 ± 3 a	78 ± 14 a	66 ± 10 ab	8 ± 5 d	96 ± 3 a	82 ± 8 a	68 ± 5 ab	54 ± 8 a	22 ± 8 cd

* Data from Yang et al. [28]. ** Means in the same column followed by the different letters differ significantly (*p* < 0.05) in ANOVA and Tukey’s tests. PR was subjected to an arcsine square-root transformation before ANOVA and Tukey’s tests.
